# Biocontrol of *Xyleborus affinis* (Curculionidae: Scolitinae) Females and Progeny by *Beauveria bassiana* (Hypocreales: Cordycipitaceae) in a Sawdust Artificial Diet Model

**DOI:** 10.3390/insects14050477

**Published:** 2023-05-19

**Authors:** Jesús E. Castrejón-Antonio, Patricia Tamez-Guerra, Nohemi García-Ortiz, Facundo Muñiz-Paredes, Juan Carlos Sánchez-Rangel, Roberto Montesinos-Matías

**Affiliations:** 1 Facultad de Ciencias Biológicas y Agropecuarias, Universidad de Colima, Autopista Colima-Manzanillo km 40, La Estación, Tecomán C.P. 28930, Colima, Mexico; 2 Facultad de Ciencias Biológicas, Departamento de Microbiología e Inmunología, Universidad Autónoma de Nuevo León, Av. Pedro de Alba S/N, Cd. Universitaria, San Nicolás de los Garza C.P. 66455, Nuevo León, Mexico; 3 Centro Nacional de Referencia de Control Biológico, km 1.5, Carretera Tecomán-Estación FFCC, Col. Tepeyac, Tecomán C.P. 28110, Colima, Mexico

**Keywords:** ambrosia beetles, entomopathogenic fungus, laid eggs, sawdust diet, conidial attachment

## Abstract

**Simple Summary:**

Ambrosia beetles may constitute a serious problem in natural and agroecosystems. The use of entomopathogenic fungi has been proposed for their biocontrol. However, beetles’ cryptic behavior requires particular bioassays to determine the true impact of fungi on adults and their progeny. In this study, we evaluated the effect of exposing adult females of *Xyleborus affinis* and their progeny to *Beauveria basssiana* in artificial diet bioassays. We demonstrated that the fungi biological activity on *X. affinis* adults and their progeny was limited to high doses of conidia suspension, since insect drilling behavior removes infective units.

**Abstract:**

The ambrosia beetle *Xyleborus affinis*, recently reported affecting avocado trees in Mexico, represents one of the most widespread insects worldwide. Previous reports have shown that *Xyleborus* genera members are susceptible to *Beauveria bassiana* and other entomopathogenic fungus strains. However, their effect on borer beetles’ progeny has not been fully investigated. The aim of the present study was to determine the insecticidal activity of *B. bassiana* on *X. affinis* adult females and their progeny in an artificial sawdust diet bioassay model. The *B. bassiana* strains CHE-CNRCB 44, 171, 431, and 485 were individually tested on females at concentrations ranging from 2 × 10^6^ to 1 × 10^9^ conidia mL^−1^. After 10 d of incubation, diet was evaluated to count laid eggs, larvae, and adults. Insect conidia loss after exposure was determined by attached conidia to each insect after 12 h of exposure. The results showed that females’ mortality ranged between 3.4% and 50.3% in a concentration–response manner. Furthermore, we did not observe statistical differences among strains at the highest concentration. CHE-CNRCB 44 showed the highest mortality at the lowest concentration and reduced larvae and laid eggs at the highest concentration (*p* < 0.01). Strains CHE-CNRCB 44, 431, and 485 significantly decreased larvae, as compared with the untreated control. After 12 h, up to 70% of conidia was removed by the effect of the artificial diet. In conclusion, *B. bassiana* has the potential to control *X. affinis* adult females and progeny.

## 1. Introduction

Ambrosia beetles are a group of wood-boring beetles that play an important role in wood degradation processes in ecosystems [1]. However, some species may become serious pests [2,3]. In this regard, *Xyleborus* species are considered dangerous pests in North America [4]. For instance, the red bay ambrosia beetle *Xyleborus glabratus* (Eichhoff) (Coleoptera: Curculionidae) was introduced to the USA from India and recognized as a serious problem to Lauraceae plants biodiversity, including avocado (*Persea americana* L.) [5]. These beetles transmit the fungus *Raffaelea lauricola* T. C. Harr., Fraedrich and Aghayeva [6,7], which causes laurel wilt disease. 

*Xyleborus affinis* represents one of the most widespread ambrosial beetles on the American continent [8]. Although it is not originally recognized as a phytosanitary issue, recent reports showed its harmful effects on avocados in Mexican orchards [9,10]. Laboratory assays conducted in Florida, USA, recognized this species as one potential carrier of *R. lauricola* [11]. In addition, some reports have shown the wide distribution of *X. affinis* and host plant abundance in countries such as Mexico, which facilitates *R. lauricola* propagation, negatively affecting the economy and ecology [12].

The introduction, establishment, and rapid propagation of exotic ambrosial beetle species and the impact of native beetles have prompted entomopathogenic fungi (EPF) biocontrol research [13]. The use of EPF represents an alternative to chemical pesticides, some of them prohibited for application, in agroecosystems such as avocado orchards [14]. In this regard, laboratory and field experiments have demonstrated the susceptibility of *X. glabratus*, *Xylosandrus crassiusculus* Motschulsky (Coleoptera: Curculionidae), and *Xylosandrus germanus* Blandford (Coleoptera: Curculionidae) to *Beauveria bassiana* (Bals.-Criv.) Vuill. (Hypocreales: Clavicipitaceae) and *Metarhizium brunneum* (Hypocreales: Clavicipitaceae) [13,15,16,17,18]. *Beauveria bassiana* is a promising fungus for the control of ambrosial beetles. Several strains of *B. bassiana* infect more than 12 species of bark beetles [2,19,20,21]. In this regard, it has been reported that the highest mortality and mycosis percentage against *X. glabratus* was caused by a *B. bassiana*-based commercial product called Botanigard^®^ES, as compared with other fungal products tested, under laboratory conditions. 

The boring behavior and cryptic habits of *Xyleborus* species must be considered when using EPF as control agents. These habits may limit the insecticidal effect of fungi, especially when median survival time (MST) for *X. glabratus* adults treated with *B. bassiana* ranges from three to seven days [13], whereas for *X. affinis*, the MST_50_ ranges from five to ten days after emerging from the pupa [22]. During these periods, it is essential to determine whether the progeny is affected by horizontal transfer of the infective units or if they remain exclusively in adults [15]. Although the phytosanitary risk of *X. affinis* is recognized, the information regarding the susceptibility of its progeny to *B. bassiana* is scarce. The aim of the present study was to evaluate the biological control of four *B. bassiana* strains on *X. affinis* adult females and their progeny in an artificial diet bioassay model, which allows this study to simulate the interaction of infested insects with the fungus considering the insect’s cryptic behavior.

## 2. Materials and Methods

### 2.1. Xyleborus affinis Origin and Molecular Identification

To establish *X. affinis* colony under laboratory conditions, adult beetles were collected in Comala, Colima, Mexico (19.45965° N, −103.65603° W), from avocado trees showing signs of decay and wilt from ambrosia beetle infestation. Authorization to collect beetles was granted by the *Comité Estatal de Sanidad Vegetal de Colima*. Adults were kept in plastic conical 50 mL tubes and fed on artificial avocado sawdust diet (15 g sawdust avocado, 4 g agar, 2 g sucrose, 1 g casein, 1 g corn starch, 1 g of brewer’s yeast, 0.2 g Wesson’s salt mix, 0.07 g tetracycline, and 100 mL of distilled water), placing five insects per tube and incubating them at 25 ± 2 °C and 65 ± 5% relative humidity for 4 wk. 

Molecular testing was conducted to confirm the identity of isolate IE233 (Xa3) as *X. affinis.* Genomic DNA extraction was performed by the HotSHOT method [23], whereas we used previously reported primers for the sequencing of a cytochrome c oxidase subunit I gene fragment (COX1) and elongation factor 1-alpha (EF-1α) [24]. PCR reactions were achieved in a Veriti Thermal Cycler (Thermo Fisher Scientific, Waltham, MA, USA), using the Platinum Taq DNA polymerase (Thermo Fisher Scientific), following the manufacturer´s instructions. PCR cycle for the COX1 gene fragment was as follows: 94 °C, 2 min; 40 cycles (94 °C, 30 s; 46 °C, 30 s; 72 °C, 1 min); and 72 °C, 5 min. For EF-1α, PCR profile consisted of 94 °C, 2 min; 16 cycles (94 °C, 30 s; 54 °C to 44 °C, 30 s; 72 °C, 1 min); 26 cycles (94 °C, 30 s; 44 °C, 30 s; 72 °C, 1 min); and 72 °C, 5 min. PCR products were purified with the Wizard SV Gel and PCR Clean-Up System kit (Promega, Madison, WI, USA) and sequenced by Macrogen (Seoul, South Republic of Korea). Sequences were edited with BioEdit 7.0.5.1 and deposited in the GeneBank (NCBI), with accession numbers MW592743 and MW590315. Identified specimens were placed in an Entomophagous Insect Collection (CIE) (https://www.gob.mx/senasica/documentos/coleccion-de-insectos-entomofagos accessed on 12 February 2019). For bioassays, 2-week-old female beetles from the established colony were used.

### 2.2. Beauveria Bassiana Strains Source

The *B. bassiana* strains CHE-CNRCB 44, 171, 431, and 485 from the *Colección de Hongos Entomopatógenos* (CHE) of the *Centro Nacional de Referencia de Control Biológico* (CNRCB) (https://www.gob.mx/senasica/documentos/coleccion-de-hongos-entomopatogenos accessed on 15 October 2022), located in the state of Colima, Mexico, were used for bioassays (Table 1). Strains were selected from a previous study based on their growth profile and virulence factors, related to the insect infection process [22].

All strains were stored on silica gel at 5 °C, until grown on Petri dishes containing Sabouraud dextrose agar (SDA), supplemented with 1% yeast extract (SDAY), which is commonly used for entomopathogenic fungi culture and assays, and incubated at 27 °C ± 2 °C in darkness.

### 2.3. Effect of Beauveria bassiana on Xyleborus affinis Adults’ Mortality and Offspring Impact

*Beauveria bassiana* strains were cultured on Petri dishes containing SDA, supplemented with 1% SDAY at pH 5.6, and incubated at 27 °C ± 2 °C in darkness [25,26]. After 20 d, conidia were harvested in a 0.05% Tween 80 solution and mixed in a vortex at maximum speed. This aqueous suspension was known as the mother solution (MS).

Conidia viability was determined by transferring 10 μL of MS to a conical microtube containing 990 mL of 0.05% Tween 80, after which it was vortexed at maximum speed for 1 min, and 10 mL of the resulting aqueous suspension were cultured in a 17 mm diameter disk of SDAY medium at 27 °C ± 2°C for 18 h. After incubation, the disk was transferred onto a coverslip and a drop of blue lactophenol (20 g of phenol, 20 g of lactic acid and 40 g of glycerol mixed in 20 mL distilled water) was added and covered with a coverslip. The percentage of viable propagules was determined by counting 100 conidia (germinated or not), using an optical microscope with a × 40 objective. Conidia were counted as viable if germ tube length was twice the size of the conidia’s diameter. Conidia suspensions were prepared at 2 × 10^6^, 1 × 10^7^, 1 × 10^8^, and 1 × 10^9^ viable conidia mL^−1^.

*Xyleborus affinis* female adults were subjected to the following 10 sec washing series: 0.05% Tween 80 solution, 70% ethanol, 0.05% Tween 80 solution, 0.1% sodium hypochlorite, and 0.05% Tween 80 solution. Insects were allowed to dry for 20 min on sterile filter paper (Whatman #4) and separated in groups of 10 in sterile glass 150 mm × 60 mm Petri dishes.

Inoculation of *Beauveria bassiana* strains was performed by immersing 20 insects in 30 mL of conidia suspensions at 2 × 10^6^, 1 × 10^7^, 1 × 10^8^, and 1 × 10^9^ viable conidia mL^−1^ in conical tubes. Insects were gently mixed for 10 sec and individually transferred to a glass tube with an artificial diet [22]. Each treatment consisted of one of the four tested concentrations and one control without conidia, inoculating 10 insects/assay in triplicate. Control insects were submerged in a sterile 0.05% Tween solution. Tubes were then incubated in darkness at 25 °C ± 2 °C for 10 d, after which each tube was broken to completely remove the diet and to count laid eggs, larvae, and adults. To evaluate fungal infection, dead individuals were placed in a humidified chamber, incubated at 25 °C ± 2 °C, and examined after 5 d to 7 d.

### 2.4. Conidial Removal from Beauveria bassiana-Inoculated Beetles

Conidia removal by the insect was determined after exposing *Xyleborus affinis* females to *B. bassiana* CHE-CNRCB 44 strain at a concentration of 1 × 10^9^ conidia mL^−1^. After exposure, beetles were transferred to 2 mL Eppendorf tubes with diet for 12 h. For the evaluation at time 0, insects were immediately placed in tubes without diet. Insects were removed from tubes with or without diet and individually transferred to 1.5 mL Eppendorf tubes containing 150 μL of 0.05% Tween 80. Tubes were then gently shaken for one minute and 10 μL was diluted to 1:1000, after which 30 µL was placed in a selective medium for *B. bassiana* (SMBb) (39 g L^−1^ potato dextrose agar; BD Bioxon, Guadalajara, Mexico), supplemented with 2.5 g L^−1^ bacteriological agar (BD Bioxon Mexico), 80 mg L^−1^ tetracycline (Sigma-Aldrich, St. Louis, MO, USA), 25 mg L^−1^ cycloheximide (Sigma-Aldrich), 80 mg L^−1^ streptomycin (Sigma-Aldrich), 80 mg L^−1^ penicillin (Sigma-Aldrich), and 1 mg L^−1^ benomyl (Robust R; Promotora Técnica Industrial, Morelos, Mexico) [26,27,28]. The inoculum was distributed using Drigalski rods, and Petri dishes were incubated at 25 °C ± 2 °C for 72 h, after which colony-forming units (CFU) were determined. A control group was kept for 12 h in a humid chamber after exposure. All bioassays were performed in triplicate.

### 2.5. Data Analysis

To establish statistically significant (*p* ≤ 0.05) differences, eggs, number of larvae, and adult mortality percentages for each treatment were transformed by (√ (x)) and arcsen (√ (p/100)), using an ANOVA with the Tukey-b multiple comparison mean test. To correct mortality from collected data against control treatment, Abbott’s transformation was used [28,29], which resulted in a <5% adjustment. Statistical analysis was performed using the SPSS V.20 software (SPSS Inc., Chicago, IL, USA).

## 3. Results

### 3.1. Xyleborus affinis Molecular Identification

The analysis of COX1 sequences via BLAST (NCBI) showed a 99.82% identity with *X. affinis* isolate AFF20MX [30] (accession number: KP941206.1). Furthermore, the EF-1α sequence showed 99.78% identity with the *X. affinis* isolate [23] (accession number: AF186688.2).

### 3.2. Xyleborus affinis Females’ Mortality

**Figure 1:**
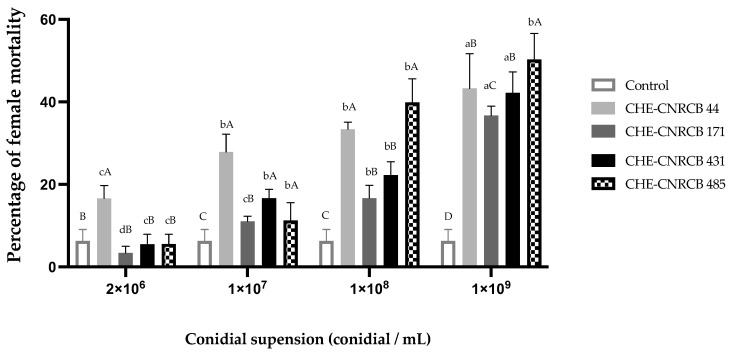
Mortality of *Xyleborus affinis* adult females inoculated with *Beauveria bassiana* strains in artificial diet. Same lowercase letter above bars indicates no significant (*p* > 0.05) difference between same strain, under different conidial suspensions. Same uppercase letter above the bars indicates no significant (*p* > 0.05) difference between same conidial aqueous suspension with different *B. bassiana* strains.

**Figure 2:**
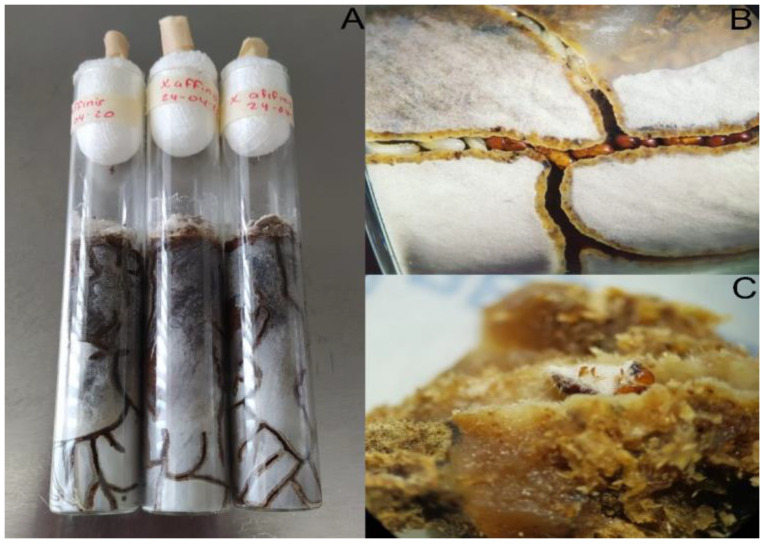
*Xyleborus affinis* colonies established in artificial diet (**A**), adult females for bioassays (**B**), and *B. bassiana*-mycosed adults (**C**).

After 10 d, mortality in female adults ranged from 3.4% to 50.3% in a concentration–response manner (Figure 1). We did not find statistically significant (F = 2.646; *p* = 0.12) differences among strains exposed to the highest concentration of conidia (1 × 10^9^ conidia mL^−1^). At the lowest concentration (2 × 10^6^ conidia mL^−1^), CHE-CNRCB 44 strain showed the highest activity, compared with all other tested strains (*p* < 0.01), resulting in 16.6% mortality. Mortality control was 6.3% ± 2.8%. Some mycosed insects (showing aerial mycelium) from fungi-treated insects were found on gallery entries (Figure 2C).

### 3.3. Beauveria bassiana Effect on Xyleborus affinis Offspring

**Figure 3:**
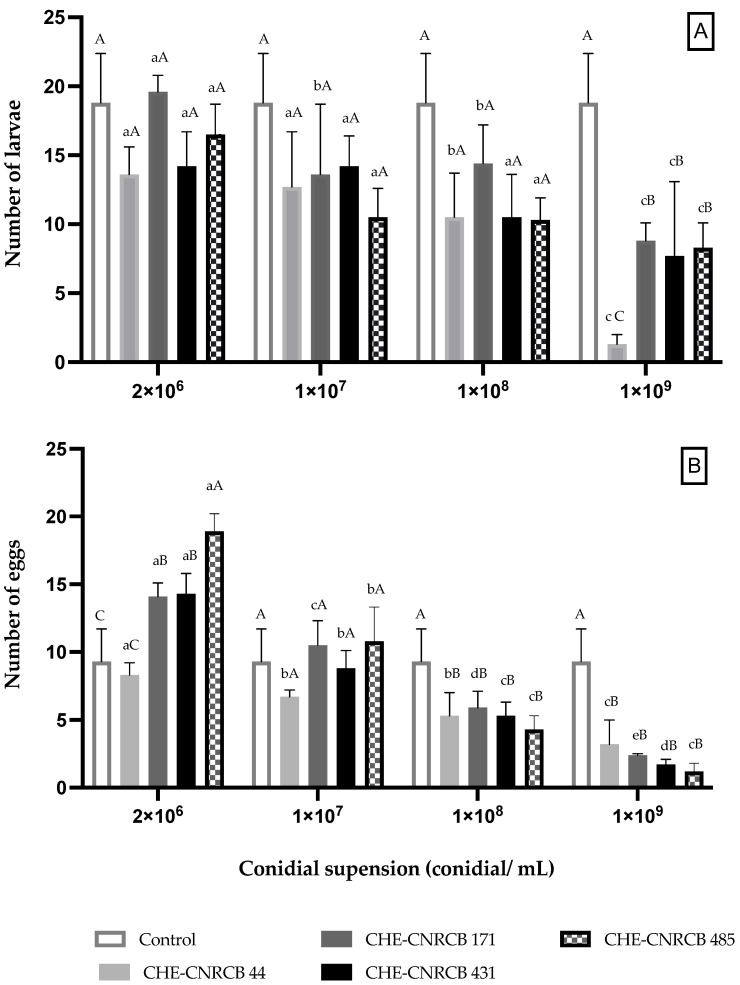
Effect of *Beauveria bassiana* strains on *Xyleborus affinis* larvae (**A**) and eggs (**B**). Same lowercase letter on bars indicates no significant (*p* > 0.05) difference between same strain under different conidial suspensions. Same uppercase letter on bars indicates no significant (*p* > 0.05) difference between same conidial aqueous suspensions.

A differential effect of *B. bassiana* strains on *X. affinis* offspring was observed (Figure 3A). At the highest conidia concentration (1 × 10^9^ conidia mL^−1^), the larval population was significantly reduced (53.2% to 93.09% reduction) after applying the CHE-CNRCB 171 and CHE-CNRCB 44 strains, respectively, as compared to the control group. 

Laid eggs were significantly reduced by all strains applied at 1 × 10^9^ conidia mL^−1^ (65.59% to 87.09% reduction), compared with the control (Figure 3B). Inoculated females laid significantly more eggs, as compared with the control, among treatments applying low conidia concentration (2 × 10^6^ conidia mL^−1^).

### 3.4. Conidial Removal from Beauveria bassiana-Inoculated Beetles

**Figure 4:**
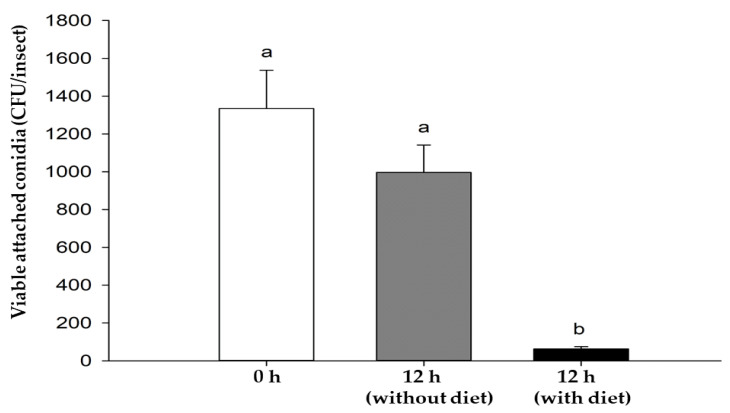
Viable conidia adhered to the cuticle of *Xyleborus affinis* females (CFU/insect) after inoculating 1 × 10^9^ conidia mL^−1^ of *Beauveria bassiana* CHE-CNRCB 44 strain. White bar represents conidia attached at time 0 h. Grey bar represents conidia attached to beetles after 12 h without diet (gray bar), and after 12 h with diet. Same letter above the bars indicates no significant (*p* > 0.05, Tukey-b) difference.

We observed no significant (*p* = 0.2387) decrease in adhered conidia after 12 h of inoculation of *X. affinis* females with the highest concentration of the *B. bassiana* CHE-CNRCB 44 strain. However, when the insects were placed in the diet immediately after being exposed, a significant (*p* < 0.001) 95% loss of the initially attached conidia was detected after 12 h of inoculation (Figure 4).

## 4. Discussion

Despite extensive data related to EPF’s potential use as biological control agents to date, our study represents valuable information regarding the ambrosia beetle. Given the insects cryptic behaviour, more information is required to estimate the ecological impact of these beneficial microorganisms.

We demonstrated the effectiveness of *B. bassiana* strains against *X. affinis* females and their progeny, obtaining mortality rates from 3.5% to 50.3%, which agrees with those reported in other beetles [30,31,32]. Moreover, the concentration that showed the best biological effect was 1 × 10^9^ conidia mL^−1^, which is particularly high compared with what is traditionally evaluated in laboratory testing [13,17,18,33,34,35].

The potential to achieve an effect on ambrosia beetle progeny has a major implication in reducing damage to host trees. Even if infestation by the founding females occurred, it is possible to stop an increase in the population by breaking the biological cycle and reducing the harmful effects on plant hosts. To produce the highest effect on progeny, significant mortality is required in the founding females. For this, significantly higher doses of inoculum are required to compensate for the loss of infective units, derived from the beetles’ boring activities. This was supported by the conidia removal test with the strain CHE-CNRCB 44 (Figure 4), which showed that insects with no diet lost 25% of their infective units. We also observed a removal of 95% of conidia after placing beetles in the diet. Therefore, the diet contributes with a removal of 70% of the conidia. Until now, this effect has not been quantified but it may be considered since it was previously reported in [17], where inoculation of the insects with EPF was followed by overnight starvation in humid chambers, and insects were transferred to the artificial diet. This favors the infective process of the fungus, in addition to the fact that prolonged starvation conditions in the insect compromise its immune system, making it more susceptible to infection [34,35,36,37]. Beetles of the genus *Xyleborus* are xylomycetophagous and thus require their cultivable fungi to grow inside the host trees or, as in this type of experiment, in the diet [36,38]. The biology of the insect demands a bioassay that mimics its natural behavior. 

The exposure by immersion that was performed in this study represents a comfortable way to elucidate some aspects about the effect of EPF in adult beetles and their progeny. It is clear that if immersion in infective units shows little impact on the beetles, other forms of exposure, such as spraying or indirect exposition via contaminated surfaces, will be less efficient. This is particularly important under field conditions, where applications are not directly made on the insect but on the tree or host plants. Achieving high amounts of infective units attached to insects represents an important challenge in dealing with ambrosial beetles. EPF formulations using inverted emulsions are an alternative to increasing the number of conidia attached to insects. An inverted emulsion is achieved between the continuous phase and the dispersed phase by changing the temperature or the composition of ingredients [39]. Although the mechanism of these emulsions is still uncertain, it may be related to its oily composition, which favors conidia attachment to the insects´ cuticle [37,38,40,41,42]. In several studies, these emulsions have been reported to increase EPF infection in various insects, including ambrosial beetles [41,42,43,44,45]. However, it is important to propose studies on contaminated surfaces to determine the adherence level of fungus to target beetles and correlate it with their biological activity, as previously reported [32,34], where the MST_50_ of three strains of EPF, but not their progeny, was determined. 

Founding females did not lay eggs during the first hours of entering their host [44,46]. Therefore, the possibility that they transmit viable infective units of EPF to their progeny is considerably reduced due to friction in the galleries and the constant removal of sawdust by the females to the outside [46,47], which may include conidia. In addition, the oxygen-limited conditions and the competition with symbiotic fungi of the beetle inside the galleries may not provide favorable conditions for the germination of the few spores that may have remained attached [31,48,49,50].

Despite the observed mortality levels in our experiments, we observed a decrease in the progeny in the galleries from 23.4% to 45.2% and 53.2% to 93.1% after exposure to 1 × 10^8^ and 1 × 10^9^ conidia mL^−1^, respectively. These progeny reductions may be more related to the death of founding females than to the horizontal transmission of the fungus to the progeny. This was supported by the lack of dead immatures in the galleries. An inverse relationship was observed between the number of eggs and conidial concentration 10 d after exposure. Thus, it is possible that oviposition reduction relates to the effect of mortality on females. Although *B. bassiana* caused mortality among adult beetles, we did not observe evidence of a change in the beetle behavior that prevented oviposition in the galleries or disturbances in larval emergence or their development.

Among the marginal studies on progeny [17], *B. bassiana* Naturalist, *B. bassiana* GHA, and *M. brunneum* F52 reduced the number of eggs, larvae, and nymphs of *Xylosandrus germanus* from 61% to 96%. In addition, and contrary to our results, fungal infection in all immature stages has been reported. This is probably due to the formulation of the aforementioned commercial products (oil-based formulation), which shows a considerable improvement in the adherence of infective units to the insects’ cuticle [13,42,43,44,45,49,51]. In our study, conidia suspensions contained Tween 80 (0.05%), which is used for their dispersal, considering their hydrophobic characteristics [52]. It is very likely that this explains the observed mortality at a concentration of 1 × 10^9^ with mL^−1^, whereas other studies reported mortality at 1 × 10^6^ mL^−1^ [13].

In the present study, mycosed adults were found in galleries, usually close to the surface, a behavior that is likely observed in other arthropods after being infected with fungi [50,53]. However, we did not find larvae or eggs with signs of fungal infection, which may suggest a possible secondary infection. This infection type has been previously discussed in other investigations that found infected immature stages after applying *B. bassiana* Naturalist to control *X. crassiusculus* [13]. According to the authors, this secondary infection mechanism may be promoted by dead infected adults inside the galleries. This has been reported for different pests as a mechanism for the generation of epizootics and as a way in which the EPF remains mobile within ecosystems [31,49,51,52]. 

When analyzing the ecology and social behavior of ambrosial beetles, the probability of contact of healthy adults in the colony with dead and mycosed insects close to the surface is low. Similarly, the hierarchy of individuals within the colonies normally minimizes transmission to immature individuals, since females that clean and feed their offspring are usually younger insects that have minimal contact with galleries closer to the surface [43,44]. In any case, the most probable scenario for secondary transmission may be generated at the moment that adult females migrate in search of new hosts, although there is more than one gallery that may be used for this purpose. Therefore, in the case of the ambrosia beetle, it is important that this secondary infection mechanism is studied in more detail and under controlled conditions. 

This study demonstrated the effect of *B. bassiana* strains application on *X. affinis* females and offspring, anticipating the probable entry of *X. glabratus* to Mexico [52,53], which may affect native Lauraceae and the Hass avocado, with a potentially serious economic and ecological impact. One perspective of our work is to demonstrate the effect on adults and progeny in models of infection by contaminated surfaces, which would provide information on the minimum amount of inoculum that must be used to inoculate the insect for effective control. Data from these experiments suggest that the conidia concentration must be significantly high to increase their attachment to the insect, for which we evaluated different fungus formulations. 

## 5. Conclusions

We conclude that *Beauveria bassiana* is an EPF that may be used in the biological control of ambrosia beetles. However, when its infective units are used without formulating them with adherent agents, it becomes necessary to use high concentrations due to the cryptic behavior of the beetles. Their cryptic activity produces a considerable removal of infective units from the insect´s body in the first 12 h. In this regard, when the founding females’ mortality reaches 50%, a significant reduction in the number of eggs laid is observed. For ambrosial beetles, it is also relevant to evaluate EPF in bioassay systems that consider the biology of this insect, which would be useful for the development of biological control agents. 

## Figures and Tables

**Table 1:** Source of *Beauveria bassiana* strains.

Strains Code (CHE-CNRCB)	GenBank Accession Number ^a^	Collection Year	Host	Geographical Origin	Reference
44	MH203469.1	2012	*Hypothenemus hampei*	Colima, México	[16]
171	MH203481.1	2012	*H. hampei*	Colima, México	[16]
431	MH203492.1	2014	*H. hampei*	Colima, México	[16]
485	ON885945.1	2015	*Galleria mellonella*	Michoacán, México	Present study

^a^ Molecular identification was based on sequencing analysis of translation EF-1α (TEF exon).

## Data Availability

The data presented in this study are available upon request from the corresponding author.

## References

[1] Vega F.E., Hofstetter R.W. (2014). Bark Beetles: Biology and Ecology of Native and Invasive Species.

[2] Popa V., Déziel E., Lavallée R., Bauce E., Guertin C. (2012). The complex symbiotic relationships of bark beetles with microorganisms: A potential practical approach for biological control in forestry. Pest Manag. Sci..

[3] Smith S.M., Hulcr J., Vega F.E., Hofstetter R.W. (2015). Scolytus and other economically important bark and ambrosia beetles. Bark Beetles: Biology and Ecology of Native and Invasive Species.

[4] Haack R.A., Rabaglia R.J., Peña J. (2013). Exotic bark and ambrosia beetles in the USA: Potential and current invaders. Potencial Invasive Pest of Agricultural Crops.

[5] Hughes M.A., Smith J.A., Ploetz R.C., Kendra P.E., Mayfield A.E., Hanula J.L., Hulcr J., Stelinski L.L., Cameron S., Riggins J.J. (2015). Recovery plan for laurel wilt on redbay and other forest species caused by *Raffaelea lauricola* and disseminated by *Xyleborus glabratus*. Plant Health Progr..

[6] Fraedrich S.W., Harrington T.C., Rabaglia R.J., Ulyshen M.D., Mayfiel A.E., Hanula J.L., Eickwort J.M., Miller D.R. (2008). A fungal symbiont of the redbay ambrosia beetle causes a lethal wilt in redbay and other Lauraceae in the southeastern United States. Plant Dis..

[7] Harrington T., Fraedrich S., Aghayeva D. (2008). Raffaelea lauricola a new ambrosia beetle symbiont and pathogen on the Lauraceae. Mycotaxon.

[8] Sobel L., Lucky A., Hulcr J. (2015). An ambrosia beetle *Xyleborus affinis* Eichhof, 1868 (Insecta: Coleoptera: Curculionidae: Scolitynae). Entomol. Nematol. UF/IFAS Ext..

[9] Castrejón-Antonio J.E., Montesinos-Matías R., Acevedo-Reyes N., Tamez-Guerra P., Ayala-Zermeño M.Á., Berlanga-Padilla A.M., Arredondo-Bernal H.C. (2017). Especies de *Xyleborus* (Coleoptera: Curculionidae: Scolytinae) asociados a huertos de aguacate en Colima, México. Acta Zool. Mex..

[10] Angeles-Restrepo M., Ochoa-Ascencio S., Fernández-Pavía S., Vázquez-Marrufo G., Equihua-Martínez A., Barrieto-Priego A.F., Correa-Suarez M., Saucedo-Carabez J.R. (2019). Identificación de escarabajos ambrosiales (Coleópteros: Curculionidae) asociados a árboles de aguacate en Michoacán, México. Folia Entomol. Mex..

[11] Carrillo D., Dunca R., Ploetz R., Peña J.E. (2014). Lateral transfer of a phytopathogenic symbiont among native and exotic ambrosia beetles. Plant Pathol..

[12] Lira-Noriega A., Soberón J., Equihua J. (2018). Potential invasion of exotic ambrosia beetles *Xyleborus glabratus* and *Euwallacea* sp. in Mexico: A major threat for native and cultivated forest ecosystems. Sci. Rep..

[13] Carrillo D., Dunlap C., Avery P., Navarrete J., Dunca R., Jackson M., Peña J.E. (2015). Entomopathogenic fungi as biological control agents for the vector of the laurel wilt disease, the redbay ambrosia beetle, *Xyleborus glabratus* (Coleoptera: Curculionidae). Biol. Control.

[14] Jones M.E., Paine T.D. (2018). Potential pesticides for control of a recently introduced ambrosia beetle (*Euwallacea* sp.) in southern California. J. Pest. Sci..

[15] Kreutz J., Zimmermann G., Vaupel O. (2004). Horizontal transmission of the entomopathogenic fungus *Beauveria bassiana* among the spruce bark beetle, *Ips typographus* (Col., Scolytidae) in the laboratory and under field conditions. Biocontrol Sci. Technol..

[16] Srei N., Lavallée R., Guertin C. (2017). Susceptibility of *Dendroctonus simplex* to Hypocreales fungi: Towards the development of a biological control strategy. J. Appl. Entomol..

[17] Castrillo L.A., Griggs M.H., Ranger C.M., Reding M.E., Vandenberg J.D. (2011). Virulence of commercial strain of *Beauveria bassiana* and *Metarhizium brunneum* (Ascomycota: Hypocreales) against adult *Xylosabndrus germanus* (Coleoptera: Curculionidae) and impact on brood. Biol. Control.

[18] Castrillo L.A., Griggs M.H., Vandenberg J.D. (2013). Granulate ambrosia beetle, *Xylosandrus crassiusculus* (Coleoptera: Curculionidae), survival and brood production following exposure to entomopathogenic and mycoparasitic fungi. Biol. Control.

[19] Selvasundaram R., Muraleedharan N. (2000). Occurrence of the entomogenous fungus *Beauveria bassiana* on the shot hole borer of tea. J. Plant. Crops.

[20] Wegensteiner R., Wermelinger B., Herrmann M., Vega F.E., Hofstetter R.W. (2015). Natural enemies of bark beetles: Predators, parasitoids, pathogens, and nematodes. Bark Beetles: Biology and Ecology of Native and Invasive Species.

[21] Montesinos-Matías R., Gallou A., Berlanga-Padilla A.M., Serna-Domínguez M.G., Laureano-Ahuelicán B., Ayala-Zermeño M.A., Ordáz-Hernández A., López-Buenfil J.A., Arredondo-Bernal H.C. (2019). Characterization of Beauveria bassiana Isolates Associated with *Euwallacea* sp. nr. *fornicates* in *Populus* sp.. Southwest. Entomol..

[22] Castrejón-Antonio J.E., Tamez-Guerra P., Montesinos-Matías R., Ek-Ramos M.J., Garza-López P.M., Arredondo-Bernal H.C. (2020). Selection of *Beauveria bassiana* (Hypocreales: Cordycipitaceae) strains to control *Xyleborus affinis* (Curculionidae: Scolytinae) females. PeerJ.

[23] Serna-Domínguez M.G., Andrade-Michel G.Y., Rosas-Valdez R., Castro-Félix P., Arredondo-Bernal H.C., Gallou A. (2019). High genetic diversity of the entomopathogenic fungus *Beauveria bassiana* in Colima, Mexico. J. Invertebr. Pathol..

[24] Truett G.E., Heeger P., Mynatt R.L., Truett A.A., Walker J.A., Warman M.L. (2000). Preparation of PCR-quality mouse genomic DNA with hot sodium hydroxide and tris (HotSHOT). Biotechniques.

[25] Normark B., Jordal B., Farrell B. (1999). Origin of a haplodiploid beetle lineage. Proc. R. Soc. B Biol..

[26] Montesinos-Matías R., Ordaz-Hernández A., Angel-Cuapio A., Colin-Bonifacio Y., Garcia-Garcia R.E., Angel-Sahagun C.A. (2021). Principal component analysis of the biological characteristics of entomopathogenic fungi in nutrient-limited and cuticle-based media. J. Basic Microbiol..

[27] Fang W., Feng J., Fan Y., Zhang Y., Bidochka M.J., Leger R.J.S., Pei Y. (2009). Expressing a fusion protein with protease and chitinase activities increases the virulence of the insect pathogen *Beauveria bassiana*. J. Invertebr. Pathol..

[28] Luz C., Netto M.C.B., Rocha L.F.N. (2007). In vitro susceptibility to fungicides by invertebrate-pathogenic and saprobic fungi. Mycopathologia.

[29] Abbott W.S. (1925). A method of computing the effectiveness of an insecticide. J. Econ. Entomol..

[30] Gohli J., Selvarajah T., Kirkendall L.R., Jordal B. (2016). Globally distributed *Xyleborus* species reveal recurrent intercontinental dispersal in a landscape of ancient worldwide. BMC Evol. Biol..

[31] Castrillo L.A., Griggs M.H., Liu H., Bauer L.S., Vandenberg J.D. (2010). Assessing deposition and persistence of *Beauveria bassiana* GHA (Ascomycota: Hypocreales) applied for control of the emerald ash borer, *Agrilus planipennis* (Coleoptera: Buprestidae), in a commercial tree nursery. Biol. Control.

[32] Draganova S.A., Doychev D.D., Pilarska D.K., Takov D.I. (2017). Bioassays of entomopathogenic fungi against xylophagous insects in Bulgaria: Laboratory and field experiments. Acta Zool. Bulg..

[33] Tuncer C., Kushiyev R., Erper I., Ozdemir I.O., Saruhan I. (2019). Efficacy of native isolates of *Metarhizium anisopliae* and *Beauveria bassiana* against the invasive ambrosia beetle, *Xylosandrus germanus* Blandford (Coleoptera: Curculionidae: Scolytinae). Egypt. J. Biol. Pest Control.

[34] Reynoso-López E.A., Méndez-Hernández J.E., Ek-Ramos J., Montesinos-Matías R., Loera O. (2021). *Metarhizium robertsii* in combination with *Trichoderma asperellum* reduce the malathion doses used to control ambrosia beetles: The case of *Xyleborus affinis*. Biocontrol Sci. Technol..

[35] Adamo S.A., Davies G., Easy R., Kovalko I., Turnbull K.F. (2016). Reconfiguration of the immune system network during food limitation in the caterpillar *Manduca sexta*. J. Exp. Biol..

[36] Deans C.A., Behmer S.T., Tessnow A.E., Tamez-Guerra P., Pusztai M., Sword G.A. (2017). Nutrition affects insect susceptibility to Bt toxins. Sci. Rep..

[37] Prior C., Jollands P., Le Patourel G. (1988). Infectivity of oil and water formulations of *Beauveria bassiana* (Deuteromycotina: Hyphomycetes) to the cocoa weevil pest *Pantorhytes plutus* (Coleoptera: Curculionidae). J. Invertebr. Pathol..

[38] Bateman R.P., Carey M., Moore D.E., Prior C. (1993). The enhanced infectivity of *Metarhizium flavoviride* in oil formulations to desert locusts at low humidities. Ann. Appl. Biol..

[39] Perazzo A., Preziosi V., Guido S. (2015). Phase inversion emulsification: Current understanding and applications. Adv. Colloid Interface Sci..

[40] Batta Y.A. (2007). Biocontrol of almond bark beetle (*Scolytus amygdali* Geurin-Meneville, Coleoptera: Scolytidae) using *Beauveria bassiana* (Bals.) Vuill. (Deuteromycotina: Hyphomycetes). J. Appl. Microbiol..

[41] Batta Y.A., Rahman M., Powis K., Baker G., Schmidt O. (2011). Formulation and application of the entomopathogenic fungus: *Zoophthora radicans* (Brefeld) Batko (Zygomycetes: Entomophthorales). J. Appl. Microbiol..

[42] Batta Y.A. (2016). Invert emulsion: Method of preparation and application as proper formulation of entomopathogenic fungi. MethodsX.

[43] Avery P.B., Bojorque V., Gámez C., Duncan R.E., Carrillo D., Cave R.D. (2018). Spore acquisition and survival of ambrosia beetles associated with the laurel wilt pathogen in avocados after exposure to entomopathogenic fungi. Insects.

[44] Morales R. (1984). Estructura de los nidos y comportamiento subsocial de *Xyleborus volvulus* (Fabricius)(Coleoptera, Scolytidae). Folia Entomol. Mex..

[45] Brar G.S., Capinera J.L., McLean S., Peña J.E. (2013). Life cycle, development and culture of *Xyleborus glabratus* (Coleoptera: Curculionidae: Scolytinae). Fla. Entomol..

[46] Garza-López P.M., Konigsberg M., Gómez-Quiroz L.E., Loera O. (2012). Physiological and antioxidant response by *Beauveria bassiana* Bals (Vuill.) to different oxygen concentrations. World J. Microbiol. Biotechnol..

[47] García-Ortiz N., Tlecuitl-Beristain S., Favela-Torres E., Loera O. (2015). Production and quality of conidia by *Metarhizium anisopliae* var. lepidiotum: Critical oxygen level and period of mycelium competence. Appl. Microbiol. Biotechnol..

[48] Rodríguez-Gómez D., Marcial-Quino J., Loera O. (2015). Modulation of conidia production and expression of the gene bbrgs1 from *Beauveria bassiana* by oxygen pulses and light. J. Invertebr. Pathol..

[49] Paradza V.M., Khamis F.M., Yusuf A.A., Subramanian S., Akutse K.S. (2021). Virulence and horizontal transmission of *Metarhizium anisopliae* by the adults of the greenhouse whitefly *Trialeurodes vaporariorum* (Hemiptera: Aleyrodidae) and the efficacy of oil formulations against its nymphs. Heliyon.

[50] Shang Y., Feng P., Wang C. (2015). Fungi that infect insects: Altering host behavior and beyond. PLoS Pathog..

[51] Cheraghi A., Habibpour B., Mossadegh M.S., Sharififard M. (2012). Horizontal transmission of the entomopathogen fungus *Metarhizium anisopliae* in *Microcerotermes diversus* groups. Insects.

[52] Reyes-Villanueva F., Garza-Hernandez J.A., Garcia-Munguia A.M., Tamez-Guerra P., Howard A., Rodriguez-Perez M.A. (2011). Dissemination of *Metarhizium anisopliae* of low and high virulence by mating behavior in *Aedes aegypti*. Parasit. Vectors.

[53] Kendra P.E., Guillén L., Tabanca N., Montgomery W.S., Schnell E.Q., Deyrup M.A., Cloonan K.R. (2023). Risk assessment of Hass avocado and Mexican Lauraceae forattack by redbay ambrosia beetle (Coleoptera: Curculionidae: Scolytinae). Agric. For. Entomol..

